# Development of Hybrid Implantable Local Release Systems Based on PLGA Nanoparticles with Applications in Bone Diseases

**DOI:** 10.3390/polym16213064

**Published:** 2024-10-31

**Authors:** Maria Viorica Ciocîlteu, Andreea Gabriela Mocanu, Andrei Biţă, Costel Valentin Manda, Claudiu Nicolicescu, Gabriela Rău, Ionela Belu, Andreea Silvia Pîrvu, Maria Balasoiu, Valentin Nănescu, Oana Elena Nicolaescu

**Affiliations:** 1 Department of Instrumental and Analytical Chemistry, Faculty of Pharmacy, University of Medicine and Pharmacy, 2 Petru Rareş Street, 200349 Craiova, Dolj County, Romania; maria.ciocilteu@umfcv.ro (M.V.C.); valentin.manda@umfcv.ro (C.V.M.); 2 Department of Pharmaceutical Technique, Faculty of Pharmacy, University of Medicine and Pharmacy of Craiova, 2 Petru Rareş Street, 200349 Craiova, Dolj County, Romania; ionela.belu@umfcv.ro (I.B.); oana.nicolaescu@umfcv.ro (O.E.N.); 3 Department of Pharmacognosy & Phytotherapy, Faculty of Pharmacy, University of Medicine and Pharmacy of Craiova, 2 Petru Rareş Street, 200349 Craiova, Dolj County, Romania; andrei.bita@umfcv.ro (A.B.); 4 Department of Engineering and Management of Technological Systems, Faculty of Mechanics, University of Craiova, 1 Călugăreni Street, 220037 Drobeta Turnu-Severin, Mehedinţi County, Romania; nicolicescu.claudiu@ucv.ro; 5 Department of Organic Chemistry, Faculty of Pharmacy, University of Medicine and Pharmacy of Craiova, 2 Petru Rareş Street, 200349 Craiova, Dolj County, Romania; gabriela.rau@umfcv.ro; 6 Department of Biochemistry, Faculty of Medicine, University of Medicine and Pharmacy of Craiova, 2 Petru Rareş Street, 200349 Craiova, Dolj County, Romania; andreea.pirvu@umfcv.ro; 7 Department of Bacteriology, Virology and Parasitology, Faculty of Medicine, University of Medicine and Pharmacy of Craiova, 2 Petru Rareş Street, 200349 Craiova, Dolj County, Romania; maria.balasoiu@umfcv.ro; 8 Pharmacy Assistants Specialization, Department of Health and Motricity, Faculty of Medical and Behavioral Sciences, “Constantin Brâncuși” University of Tg-Jiu, Romania No. 4, Tineretului St., 210185 Tg-Jiu, Gorj County, Romania; valentin.nanescu@gmail.com

**Keywords:** hybrids, biopolymers, bone infection, nanoparticles, PLGA, ciprofloxacin, implantable systems

## Abstract

The current strategy for treating osteomyelitis includes surgical procedures for complete debridement of the formed biofilm and necrotic tissues, systemic and oral antibiotic therapy, and the clinical use of cements and three-dimensional scaffolds as bone defect fillers and delivery systems for therapeutic agents. The aim of our research was to formulate a low-cost hybrid nanoparticulate biomaterial using poly(lactic-co-glycolic acid) (PLGA), in which we incorporated the therapeutic agent (ciprofloxacin), and to deposit this material on titanium plates using the matrix-assisted pulsed laser evaporation (MAPLE) technique. The deposited material demonstrated antibacterial properties, with all analyzed samples inhibiting the growth of tested bacterial strains, confirming the release of active substances from the investigated biocomposite. The poly(lactic-co-glycolic acid)-ciprofloxacin (PLGA-CIP) nanoparticle scaffolds displayed a prolonged local sustained release profile over a period of 45 days, which shows great promise in bone infections. Furthermore, the burst release ensures a highly efficient concentration, followed by a constant sustained release which allows the drug to remain in the implant-adjacent area for an extended time period.

## 1. Introduction

Chronic osteomyelitis is an inflammatory condition determined mainly by a bone infection [1]. Osteomyelitis remains a challenge in Romanian orthopedic medical practice despite current medical and surgical advancement because the treatment may extend over weeks and even months [2]. Moreover, it requires a prolonged and complex management involving surgical interventions and antibiotic treatment.

*Staphylococcus aureus* has the highest incidence in orthopedic infections. It accounts for approximately 20–30% of orthopedic infections, whereas coagulase-negative staphylococci are linked with 20–40% of the cases [3]. Furthermore, *Staphylococcus aureus* adheres to the bone surface, developing an antibiotic-resistant biofilm [4].

Ciprofloxacin (CIP) is used to treat a wide range of infections due to its broad spectrum of action, which includes most pathogens. It is particularly effective against Gram-negative bacteria and less effective against Gram-positive bacteria compared to newer fluoroquinolones [5]. Ciprofloxacin is used in daily practice not only for its broad spectrum of action but also for its good availability and bone penetration [6].

Therefore, it is used in the treatment of bone and joint infections, intra-abdominal infections, respiratory tract infections, skin infections, typhoid fever, urinary tract infections, and others. In treating infections, it is used both in monotherapy and in combination with other antibiotics [7]. CIP is easily absorbed, but the absorption is usually not complete, with a bioavailability ranging between 70 and 80% [8].

CIP is administered in bone infection treatments lasting between 4 weeks and 6 months, with a cure rate of 70% of cases and a significant improvement in another 5% of cases over a follow-up period of several weeks to one year [9].

Only oral and intravenous formulations are currently available for ciprofloxacin. Following 60 to 120 min of intravenous infusion or an oral administration of 200–400 mg of ciprofloxacin, the maximum peak plasma concentration reached was 4.6 µg/mL. The half-life (the time required for the serum concentration to decrease by 50% after administration) is approximately 4–6 h [10,11]. The absorption rate is influenced by intestinal pH, with higher absorption in the duodenum and jejunum [12]. It is estimated that only 1% of ciprofloxacin reaches the infection when oral administration is employed [13]. Therefore, current formulations cannot eradicate the biofilm without severe systemic side-effects. Although CIP also has low permeability, for most antibiotics that do not belong to the fluoroquinolone class, the bone/serum concentration ratio is only 0.3–0.67 at 0.5 to 2 h [14].

Thabit et al. explain this difference by the potential binding of quinolones to calcium ions in hydroxyapatite (the bone’s inorganic matrix) [15].

Due to the high costs of treatment, multiple amputations, and prolonged morbidity in patients undergoing traditional treatment, single-stage surgical protocols have recently been developed as a result of the advancement of biodegradable biocomposites loaded with antibiotics [16].

In recent years, there has been an increasingly intensive effort to find a solution for treating bone infections that minimizes systemic toxicity while ensuring optimal concentration at the contaminated site, through controlled local release of the active substance from a biodegradable device [17]. The local release of antibiotics in osteomyelitis is a highly studied alternative to systemic administration. Moreover, local vascularization is compromised as a result of the debridement of the infected bone tissue. Therefore, ensuring a minimum inhibitory concentration is difficult to attain.

CIP, due to its broad spectrum of action against bacterial agents associated with osteomyelitis, has been frequently investigated in the study of drug delivery systems (DDSs) [18].

The sustained local delivery of antibiotics is considered advantageous because it facilitates increased patient compliance and high efficiency.

Most human tissues, such as bones, tendons, ligaments, skin, and teeth, are composites, with many constituents whose quantity, distribution, morphology, and properties determine the final behavior of the tissue or organ. This fact later allowed the synthesis of composites capable of imitating these biological tissues, imitating the mechanical behavior to restore a function of a damaged tissue [19,20].

**Figure 1:**
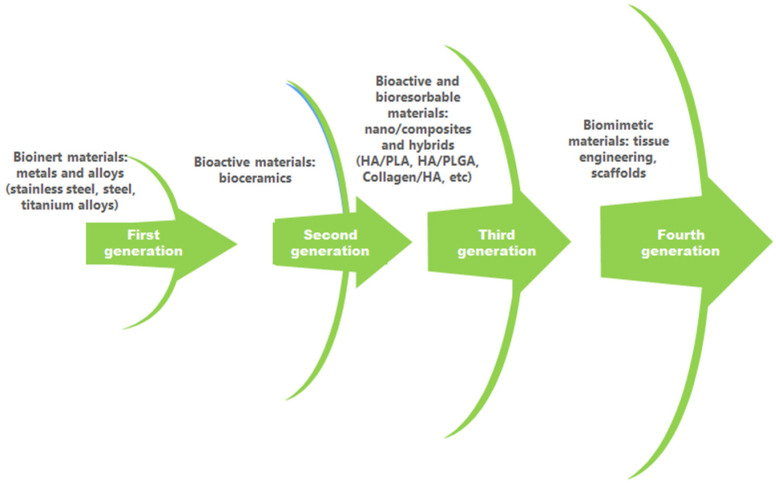
Biomaterial evolution in bone repair and regeneration.

The evolution of biomaterials in bone repair and regeneration is depicted in Figure 1.

Encapsulation in the pharmaceutical field is mainly focused on formulating drug delivery systems with fewer side-effects, better posology, sustained and controlled release of the drug, and specific tissue targeting [21].

PLGA (polylactic-co-glycolic acid) is generally considered safe for use, particularly in biomedical applications [22]. It has been widely employed in drug delivery systems, sutures, and medical implants due to its biocompatibility and biodegradability. The FDA has approved PLGA for various medical purposes, including drug delivery systems and biodegradable sutures [23]. It naturally breaks down into lactic acid and glycolic acid, which are byproducts that the body can safely metabolize and eliminate [23]. In both preclinical and clinical studies, PLGA has demonstrated low toxicity and is well tolerated by most tissues [24,25].

The aim of this study was to develop a hybrid local release system (LRS) based on poly(lactic-co-glycolic acid) (PLGA) and CIP in order to obtain a sustained and prolonged local release and to deposit it using the MAPLE technique onto titanium, obtaining an implantable material that accelerates the bone healing and regeneration process, has reduced immunoreactivity, has a lower risk of rejection or hypersensitivity reactions, and avoids the possibility of transmitting an infection. Compared to other studies, the novelty elements brought by our study are the deposition of the PLGA-CIP material on titanium disks through an innovative technique (MAPLE). In chronic cases where the infection has compromised the structural integrity of the bone, these implantable devices can provide mechanical support since they are designed with titanium (biocompatible metal), combined with PLGA and CIP, to offer both structural reinforcement and therapeutic benefits [26,27,28].

## 2. Materials and Methods

All chemicals used in this study were of analytical grade. PLGA (50:50) was acquired from Sigma-Aldrich (München, Germany), and ultrapure water was purchased from LiChrosolv^®^ Merck and used in all experiments. Both CIP and polyvinyl alcohol (PVA) 8–88 (MW ~67,000 g/mol) were purchased from Sigma-Aldrich (München, Germany). Citrate buffer, ammonium hydroxide, commercial hydroxyapatite, dichloromethane (DCM), acetonitrile (ACN), acetone, and ethanol were acquired from Sigma Aldrich (München, Germany) or Merck Millipore (Darmstadt, Germany).

### 2.1. PLGA-CIP Formulation

**Figure 2:**
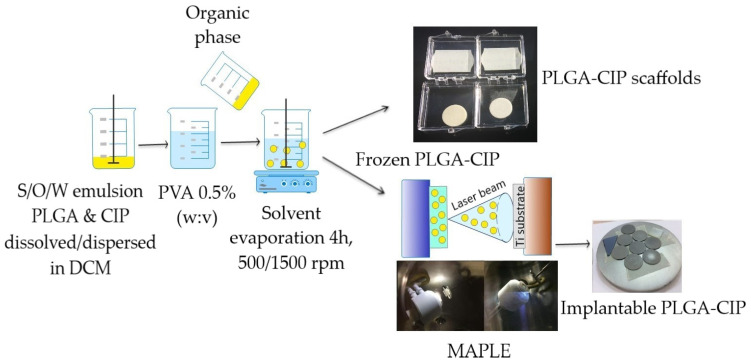
Formulation of PLGA-CIP and PLGA-CIP implantable local release systems.

PLGA-CIP nanoparticles were obtained using the solid/oil/water solvent evaporation method as follows: CIP (50 mg) was dispersed into the oily phase. The oily phase was obtained by dissolving PLGA (100 mg) into dichloromethane (9 mL DCM). A polyvinyl aqueous with 0.5% PVA as the emulsifier (*w*/*v*) was obtained separately. The oily phase was mixed at 30.000 rpm in a Heidolph Silent Crusher Vortex (Wood Dale, IL, USA). Then, it was poured over the PVA solution (600 mL 1% PVA solution) and mixed for 4 h at 500/1500 rpm. The PLGA nanoparticles were washed in triplicate and dried. In order to evaluate both the release profile and antibacterial activity, part of the obtained material was compacted in the form of disks using a press (Figure 2).

### 2.2. Obtaining Implantable PLGA-CIP LRS

The MAPLE deposition technique was used to transfer the PLGA-CIP system onto a Ti substrate. The deposition experiments were carried out using a COMPexPro 205 laser model, produced by Lambda Physik/Coherent, equipped with a KrF* excimer laser with a wavelength of 248 nm and a pulse duration of 25 ns. The pulsed laser irradiation was performed in a stainless steel chamber equipped with pressure control and air evacuation equipment (rotary pump, model Alcatel SD 2033). For target preparation, n-hexane was chosen as the solvent, as it ensures an adequate redispersion of PLGA nanoparticles and is also highly volatile. Dispersions of 1% PLGA-CIP nanoparticles in 99% n-hexane (5 mL) were prepared. To make the dispersion homogeneous, an ultrasonic bath and a vortex mixer were used. The suspension was then poured into a copper device with a diameter of 3 cm and frozen in liquid nitrogen (−196 K). The resulting target was introduced into the reaction chamber and then subjected to laser irradiation.

Grade 4 titanium substrates were cleaned in a mixture of ethanol, acetone, and water in an ultrasonic bath and then introduced into the reaction chamber. The deposition conditions of PLGA-CIP on the Ti substrate are shown in Table 1.

### 2.3. Fourier Transform Infrared (FTIR) Spectroscopy

FTIR analysis was carried out using an Avatar Nicolet iN 10 spectrophotometer in KBr pellets within the range of 4000–400 cm^−1^.

### 2.4. Loading Efficiency

Loading efficiency was determined using a Thermo Finnigan Surveyor HPLC system equipped with a diode array detector (DAD). Separation was achieved on a C18 reversed-phase column Hypersil Gold (250 mm length, 4.6 mm inner diameter). The mobile phase was a mixture of 20 mM citrate buffer and acetonitrile with a flow rate of 1 mL∙min^−1^. All experiments were conducted at room temperature. The linearity range for CIP was between 125 and 20,000 ng/mL, with a correlation coefficient of 0.99993 with the linear regression equation A/(mAu*min) = 1014 c/(ng/mL)-177196, where A is the peak area (mAu*min) and c is the concentration (ng/mL).

CIP extraction from PLGA-CIP nanoparticles: 10 mg of PLGA-CIP was dissolved in 3 mL of DCM. An amount of 2 mL of water was added to the solution and the pH was adjusted to 11 with ammonium hydroxide. The sample was ultrasonicated for 20 min and centrifuged at 10,000 rpm for 10 min. The supernatant was extracted and mobile phase was added to make up 5 mL. Finally, 20 µL was injected into the system.

LE% was calculated using the following formula:

$$\mathrm{Loading}\;\mathrm{efficiency}\;\left(\%\right)=\frac{\mathrm{CIP}\;\left(\mathrm{mg}\right)}{\mathrm{PLGA}-\mathrm{CIP}\;\;\mathrm{mass}\;\left(\mathrm{mg}\right)}\times 100\%$$

### 2.5. Dynamic Light Scattering (DLS) Particle Size Distribution Analysis

Volume and number size distributions were determined by DLS using a Brookhaven 90 Plus particle size analyzer equipped with a BI-ZETA instrument and a solid-state laser (15 mV; scattering angle: 15°, 90°) used in the range of 1–6000 nm. All measurements were conducted at 25 °C. The nanoparticles were resuspended in ultrapure water and ultrasonicated for 20 min. The zeta potential was measured in the following range: −150 to +150 mV; size range: 10 nm to 30 µm; accuracy: ±2%; repeatability: ±2%; laser: 35 mW solid-state laser, red (660 nm wavelength); and sample volume: 1.5 mL.

### 2.6. Scanning Electron Microscopy (SEM) Analysis

The analyses were carried out using a Hitachi Ultra-High-Resolution FE-SEM SU8230. The nanoparticles were dispersed in 1% n‒hexane. The nanoparticle suspension was added to aluminum plates coated with a double carbon film. The solvent was evaporated at room temperature and the samples were evaluated by SEM.

### 2.7. In Vitro Release Profile

Scaffolds composed of PLGA-CIP and commercial hydroxyapatite (HA) were obtained in order to evaluate the release profile of CIP as follows:

- PLGA-CIP (1500 rpm);
- PLGA-CIP:HA (*w*:*w*) 25:75;
- PLGA-CIP:HA (*w*:*w*) 50:50;
- PLGA-CIP:HA (*w*:*w*) 75:25;
- CIP:HA (*w*:*w*) mechanical mixture;

The scaffolds were placed in tightly closed containers and 5 mL of ultrapure water was added. Then, they were incubated at 37 °C. The dispersion was mixed at 3000 rpm. Then, 1 mL of eluted drug medium was removed for quantification by HPLC-DAD, at various time points (3 h, 6 h, 24 h, 48 h, 72 h, 120 h, 7 days, 14 days, 21 days, 30 days, 45 days). This volume was replaced with fresh water to prevent sink conditions.

#### In Vitro Release of Implantable PLGA-CIP LRS

The implantable PLGA-CIP LRS was added to 5 mL of ultrapure water and the vials were kept in the oven at 37 °C. At regular time intervals (3 h, 6 h, 24 h, 48 h, 72 h, 120 h, 7 days, 10 days), the solutions were stirred at 3000 rpm and then a volume of 1 mL of solution was taken for quantitative determination of CIP by HPLC-DAD. The sampled volume was replaced with ultrapure water to avoid saturation of the CIP solution.

### 2.8. Antibacterial Activity

Nutrient agar (Mueller–Hinton) was evenly poured into 100 mm diameter Petri dishes, forming a 4 mm thick layer. The inoculum was prepared by suspending 2−3 standard bacterial colonies in physiological saline, with the turbidity of the suspension measured nephelometrically. The culture medium had a pH of 7.3 and was appropriately formulated for the growth of the bacterial species under investigation.

The seeding process involved flooding the nutrient medium with the bacterial suspension, followed by removing the excess liquid. The inoculated plates were left to dry for 10 min at room temperature (22 °C) before placing the samples. The microorganisms tested originated from standard reference strains obtained from the Cantacuzino Institute, known to be sensitive to CIP, the antibiotic used in the study. The test disks were positioned 1.5 cm from the edge of the Petri dish. Incubation was performed for 18 h at 37 °C, with the Petri dishes placed in an inverted position.

The results were observed visually, and the diameter of the inhibition zones (in mm) induced by the test samples was measured using a graduated ruler.

### 2.9. PLGA-CIP Scaffolds

The PLGA-CIP scaffolds were evaluated by the disk diffusion method against Staphilococcus aureus (ATCC 25923) and methicillin-resistant S. aureus (ATCC 43300). PLGA-CIP samples were applied approximately 15 min after seeding, with each sample placed on the surface of the culture medium for analysis. A standard disk containing CIP was used as the reference control. Studies were performed in triplicate and the mean value was determined.

### 2.10. Implantable PLGA-CIP LRS

The diffusimetric method was used to test the sensitivity of bacteria such as Staphylococcus aureus (ATCC 25923) and methicillin-resistant S. aureus (ATCC 43300) to implantable PLGA-CIP LRS.

As a reference, the effect of implant systems coated with CIP on the reference strains was studied comparatively.

### 2.11. Statistical Analysis

All experiments were performed in triplicate for all samples, all calibration curves, and concentrations. Statistical analysis was carried out using Microsoft Office Excel 2019 (Microsoft Corporation, Redmond, WA, USA), and are expressed as the mean ± SD. *p*-values < 0.05 were considered statistically significant. Graphical figures were obtained with ConceptDraw Diagram Version 16 software.

## 3. Results

### 3.1. Fourier Transform Infrared (FTIR) Spectroscopy

**Figure 3:**
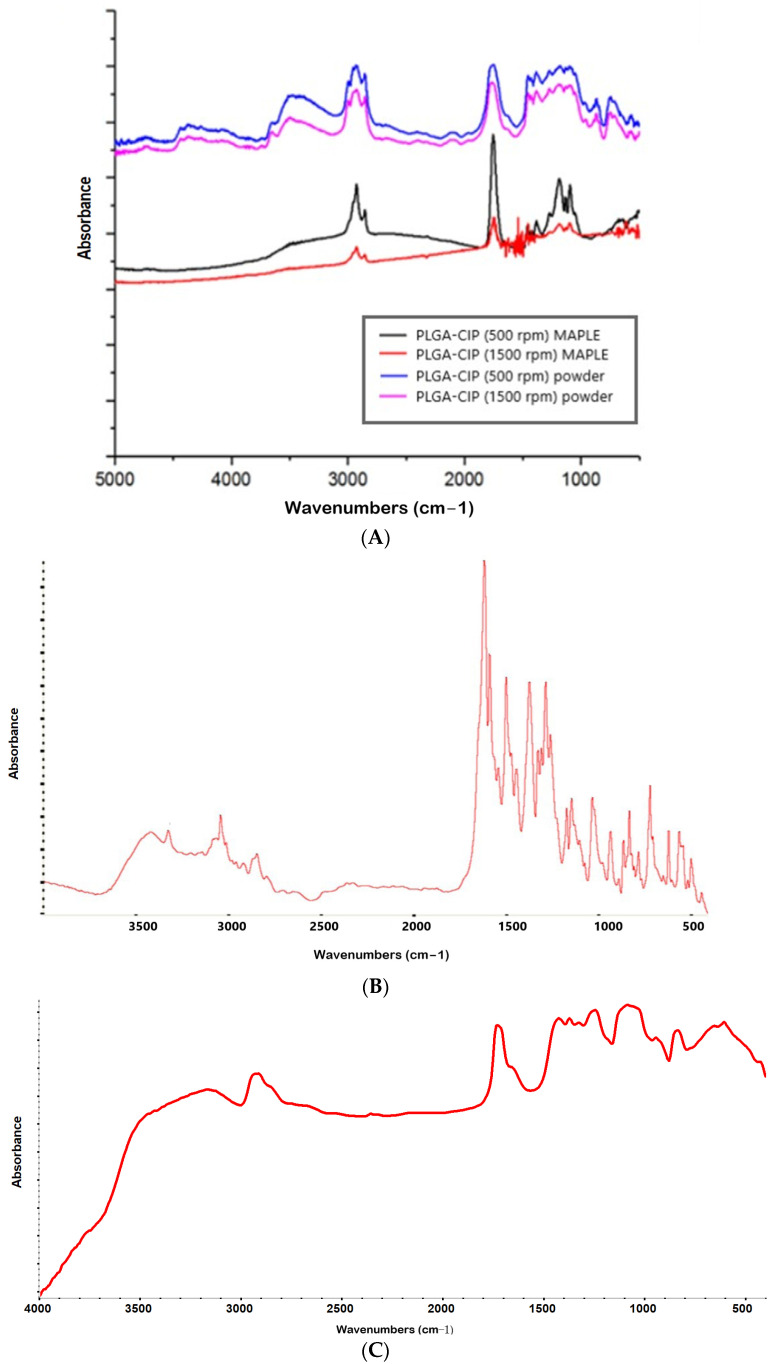
FTIR spectra of (**A**) PLGA-CIP and PLGA–CIP films deposited by MAPLE on titanium supports; (**B**) CIP; (**C**) PLGA.

The intense peak at 1747 in both PLGA and PLGA-CIP spectra is determined by the carbonyl stretching absorption (Figure 3A,C). Peaks that are characteristic of stretching vibration of the deprotonated carboxyl group in the spectrum of CIP (Figure 3B) and PLGA-CIP can be observed at 1591 cm^−1^ (asymmetric stretching) and at 1376 cm^−1^ (symmetric stretching). Although with a lower intensity, the FTIR spectra of the films deposited by MAPLE present both the characteristic peak at 1750 cm^−1^ and the peaks characteristic of the stretching vibration of the ester groups in the range of 1300−1600 cm^−1^, which suggests that the structure of the PLGA-CIP nanoparticles was not affected during the MAPLE deposition process.

### 3.2. Loading Efficiency

The loading efficiency of CIP from PLGA-CIP obtained by the solid-in-oil-in-water (S/O/W) method was 28.9% (1500 rpm) and 22.1% (500 rpm). The chromatogram for CIP determination from PLGA-CIP (1500 rpm) is presented in Figure S1A, and in S1B for PLGA-CIP (500 rpm), respectively.

### 3.3. Dynamic Light Scattering (DLS) Particle Size Distribution Analysis

**Figure 4:**
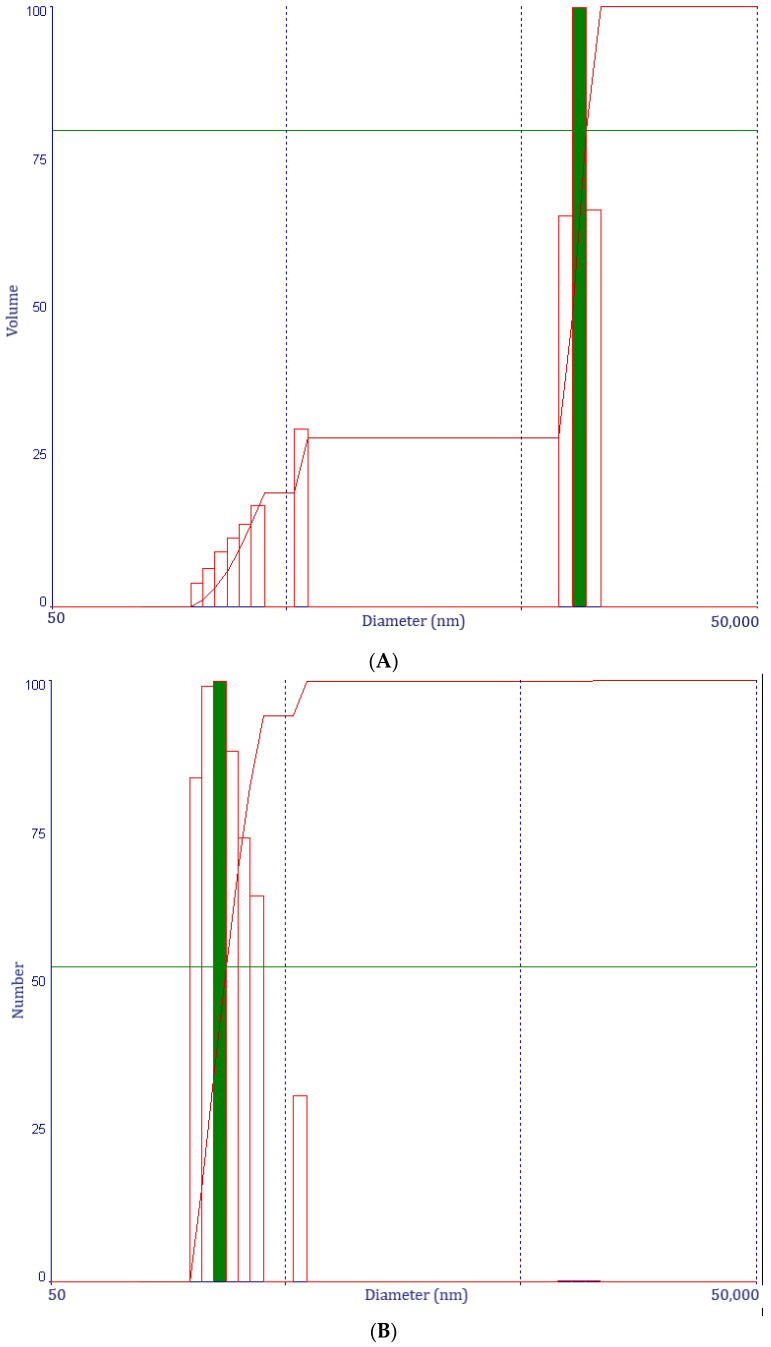

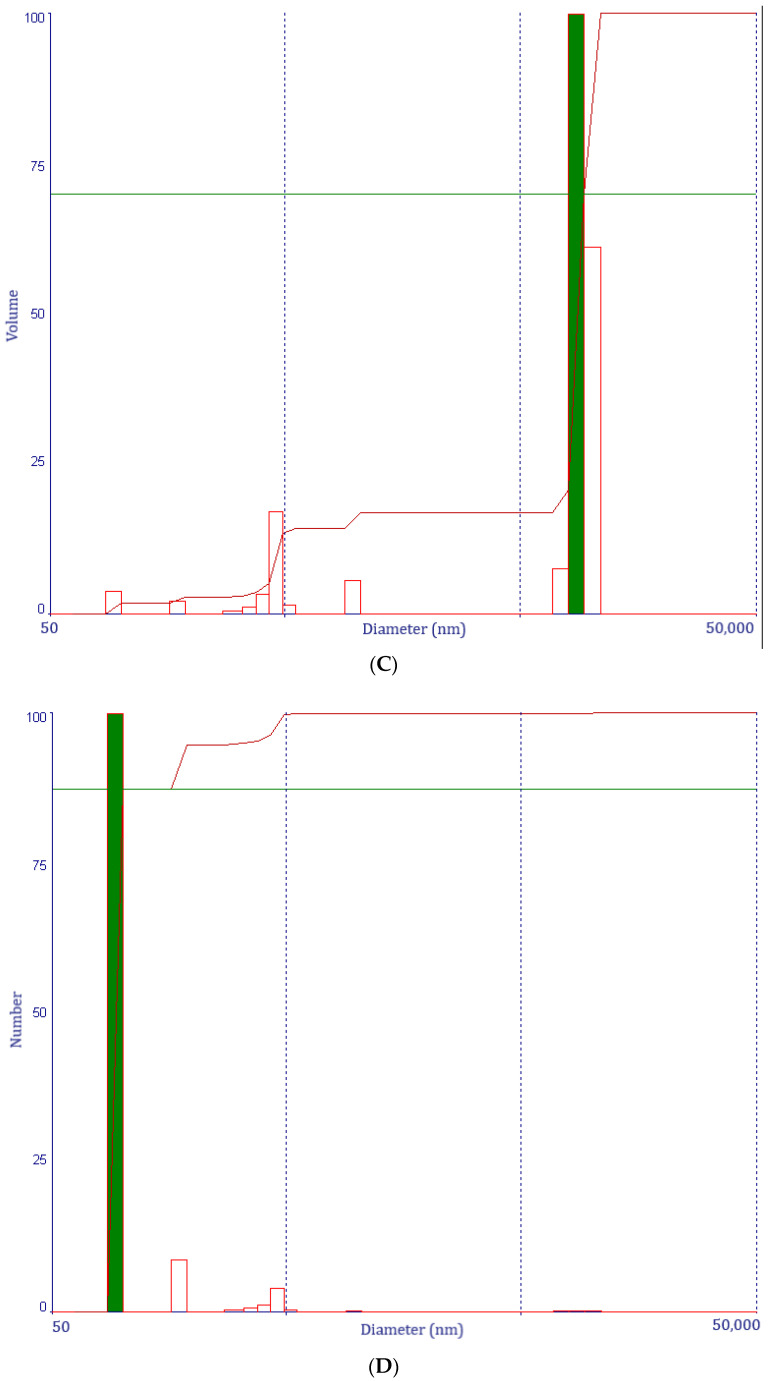
Volume distribution of PLGA-CIP (500 rpm) (**A**); number distribution of PLGA-CIP (500 rpm) (**B**); volume distribution of PLGA-CIP (1500 rpm) (**C**); number distribution of PLGA-CIP (1500 rpm) (**D**).

It was determined that for PLGA-CIP (500 rpm), the sample exhibits a bimodal volume distribution, with two particle size intervals: [206–571] nm and [7.61–10] µm, respectively (Figure 4A). The number distribution shows that only the first particle size interval remained, [206–571] nm (Figure 4B).

For PLGA-CIP (1500 rpm), a volume distribution with two particle size ranges was determined: [349–517] nm and [7.31–10] µm (Figure 4C). There is also a very small volume of particles distributed as follows: 0.02% of the total volume is around the size of 92.5 nm; 0.01% of the total volume is around the size of 172.6 nm; and 0.03% of the total volume is around the size of 961.6 nm.

In the case of the number distribution: 87% of the total number of particles are around the size of 92.5 nm; 8% are around the size of 172.6 nm; 0.9% are around the size of 349.8 nm; 0.9% are around the size of 398.4 nm; and 3.2% are around the size of 453.7 nm (Figure 4D).

The average value of the zeta potential was -40.08 mV for PLGA-CIP (1500 rpm) (Figure S2A), and −38.04 mV for PLGA-CIP (500 rpm) (Figure S2B), which is favorable for bone regeneration and osseointegration. In our opinion, there are no significant differences between the two samples in terms of zeta potential, with the stirring speed not influencing its value.

### 3.4. Morphological Aspects of PLGA-CIP

**Figure 5:**
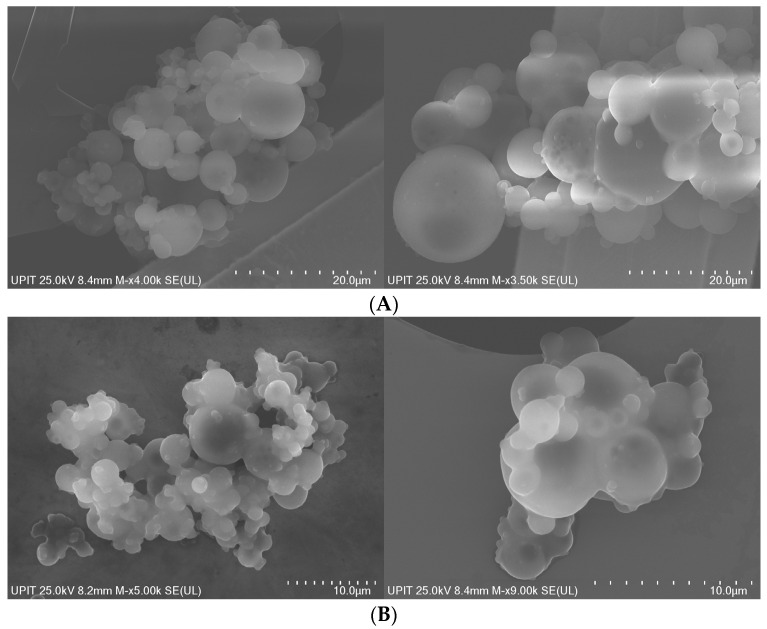
Scanning electron microscopy images of (**A**) PLGA-CIP (1500 rpm) and (**B**) PLGA-CIP (500 rpm).

The shape of poly(lactic-co-glycolic acid) (PLGA) nanoparticles obtained through the S/O/W emulsion technique is typically spherical (Figure 5). The porous spherical structure is characteristic of all PLGA materials obtained by the emulsion method and is determined by the volatilization of the organic solvent from the secondary aqueous phase.

### 3.5. In Vitro CIP Release Profile

**Figure 6:**
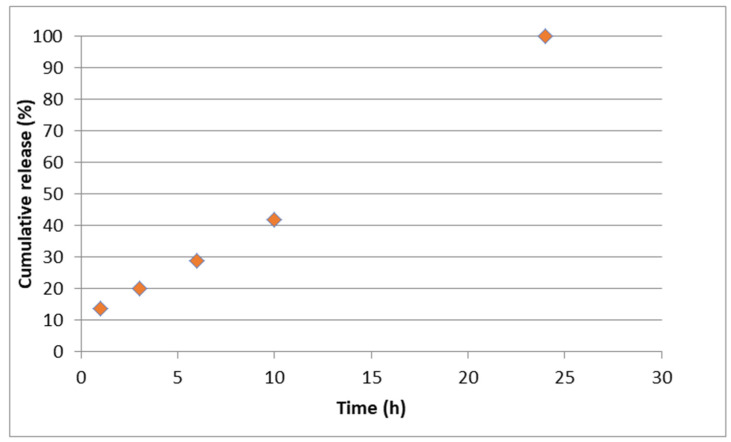
CIP release from the control sample: mechanical mixture CIP:HA (*w*:*w*) (25:75).

The in vitro ability of PLGA-CIP formulations to prolong the release of ciprofloxacin was evaluated, since from the compressed CIP in a mechanical mixture with HA, CIP release was complete within 24 h when compressed in a mechanical mixture with HA (Figure 6).

**Figure 7:**
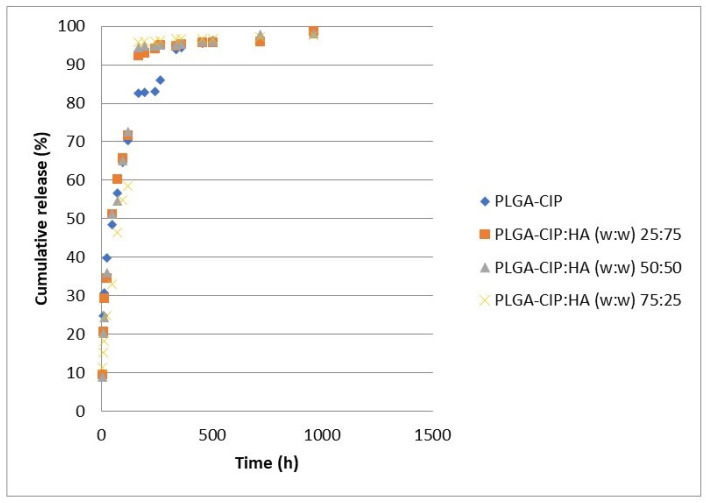
CIP release from PLGA-CIP scaffolds.

The results (Figure 7) show that the samples containing only nanoparticles and those containing a mixture of nanoparticles and hydroxyapatite exhibit a release profile divided into two stages. The first part of the curve, corresponding to the release during the first 3 days, represents a rapid burst release. In all samples, approximately 60% of the CIP is released during this phase, following first-order kinetics. Subsequently, the release follows zero-order kinetics, which is characteristic of prolonged release systems. In all cases, the release continued up to 45 days, following mixed kinetics.

### 3.6. In Vitro CIP Release Profile of Implantable PLGA-CIP LRS

**Figure 8:**
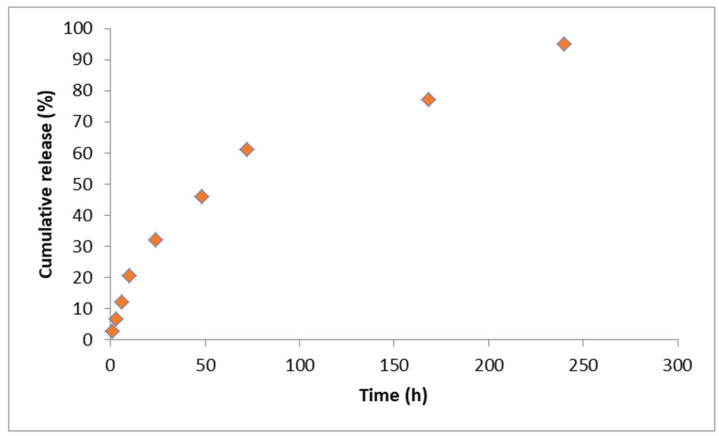
Release profile of CIP from implantable PLGA-CIP LRS.

The release profile of CIP encapsulated in PLGA and deposited by MAPLE on Ti is shown in Figure 8. Thus, in the first 48 h, approximately 60% of the CIP was released, followed by a slower release, as observed in PLGA-CIP scaffolds. In this case, CIP release occurred over a period of 10 days, which could be attributed to the small amount deposited on Ti and the fact that the nanoparticles were deposited as a thin film.

**Figure 9:**
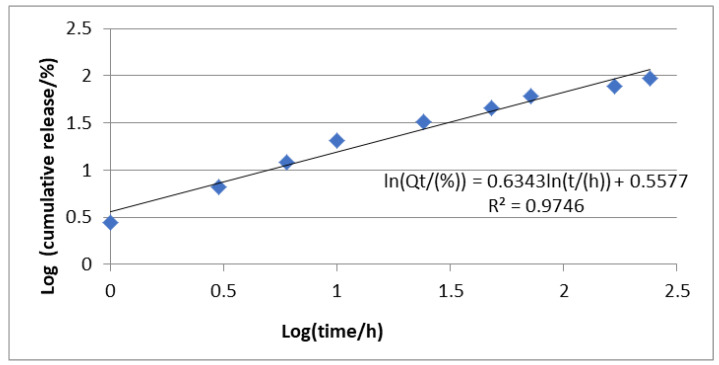
Korsmeyer–Peppas model for the mechanism of drug release.

**Figure 10:**
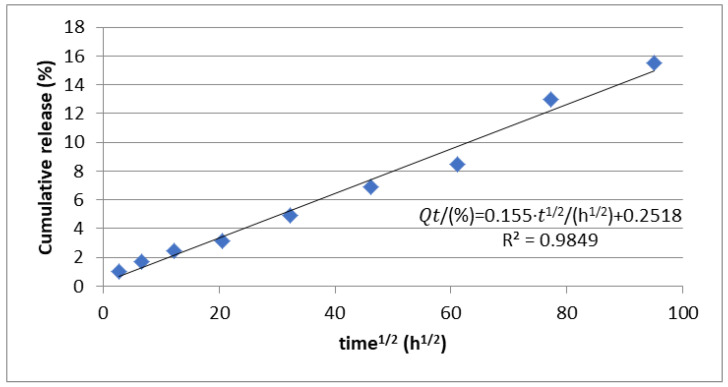
Higuchi release model or the mechanism of drug release.

The release profile was investigated using the Higuchi and Korsmeyer–Peppas (Figure 9 and Figure 10) models. Thus, following the application of the Higuchi model, a correlation coefficient of 0.98 was obtained, which suggests that the diffusion process intervenes in the release of CIP from the deposited nanoparticles. Also, after applying the Korsmeyer–Peppas model, a correlation coefficient of 0.97 was obtained, and the value of n was 0.63. Since the value of n was between 0.45 and 0.95, it is considered that in addition to diffusion, the release of CIP is also influenced by the erosion process (Figure 10).

### 3.7. Antibacterial Activity

**Figure 11:**
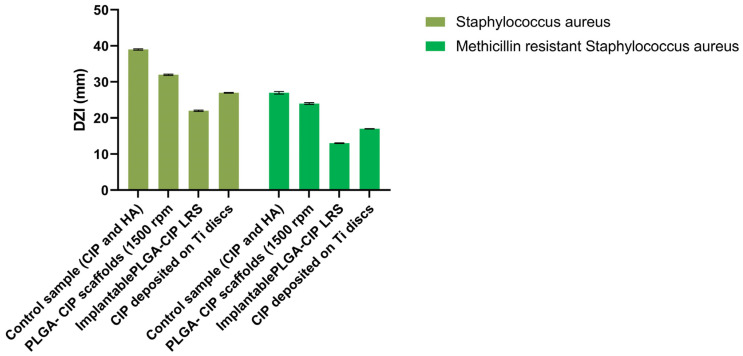
Antibacterial activity of scaffolds over tested germs.

**Figure 12:**
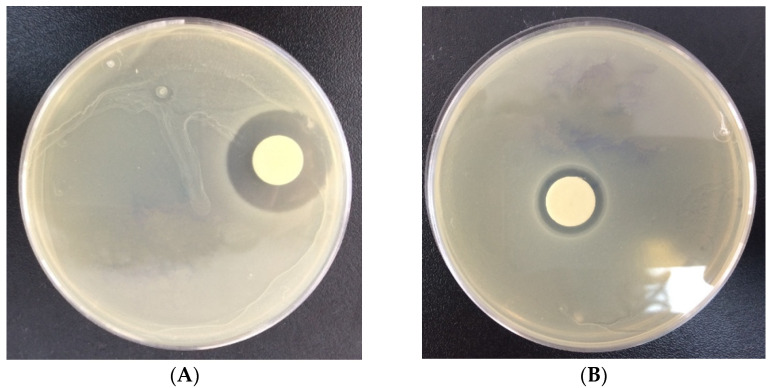
The zone of inhibition obtained for (**A**) PLGA-CIP scaffolds on Staphylococcus aureus; (**B**) PLGA-CIP scaffolds (1500 rpm) on methicillin-resistant Staphylococcus aureus using the disk diffusion agar method.

**Figure 13:**
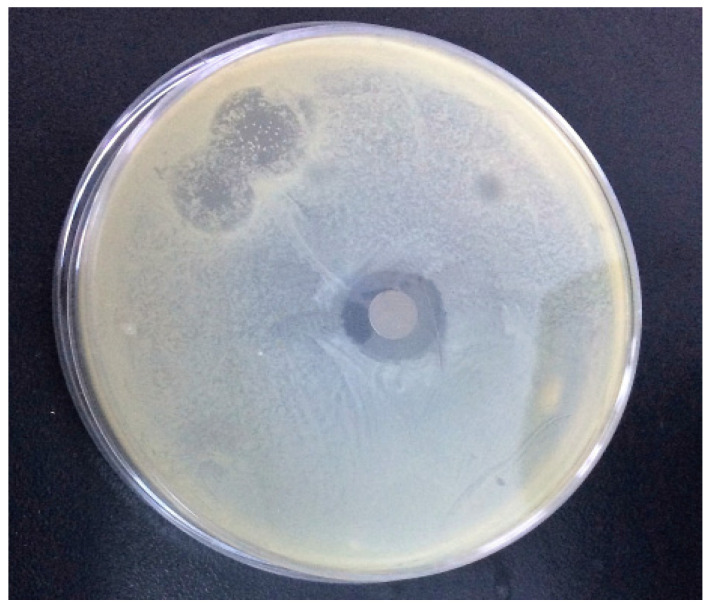
The zone of inhibition obtained for implantable PLGA-CIP LRS (1500 rpm) on *Staphylococcus aureus.*

For implantable PLGA-CIP LRS, the diameter of the inhibition zone (DZI) was 22±0.16 mm in the antibiogram performed on *Staphylococcus aureus*, and 13±0.12 mm for the antibiogram performed on methicillin-resistant *Staphylococcus aureus*, which suggests that only *S. aureus* is sensitive to the implant-type systems (Table 2, Figure 11). Encapsulated CIP gradually inhibited bacterial growth, and after 24 h, when the inhibition zones were measured (Figure 12), only 33% of the CIP had been released, according to the release profile. From the results presented earlier, it can be observed that by including CIP in PLGA nanoparticles and depositing them on implant-type delivery systems, the antibiotic’s activity against *S. aureus* is preserved (Figure 13).

## 4. Discussion

Through these hybrid implantable systems for osteomyelitis treatment, we tried to develop an advanced therapeutic approach that combines various technologies to address bone infections more effectively. Osteomyelitis, a severe infection of the bone, often caused by bacteria like *Staphylococcus aureus*, is notoriously difficult to treat due to poor vascularization and the protective biofilm produced by pathogens. Traditional treatments involve systemic antibiotics and surgical debridement, but these often fall short in fully eradicating the infection, especially in chronic cases [29].

### 4.1. FTIR Analysis

By FTIR analysis, we identified the component materials (PLGA and CIP) in the final hybrid material (PLGA-CIP). The peaks specific to ester groups identified in Figure 3A show that the polymer structure was not modified during nanoparticle preparation and deposition. Furthermore, there is no shift in the characteristic bands in both compounds, which suggests that there is no interaction between PLGA and CIP in the synthesized nanoparticles [30,31].

### 4.2. Loading Efficiency

In order to determine the loading efficiency for CIP, we used an acidic mobile phase in order to obtain symmetrical, easy-to-integrate peaks without “heading” or “tailing”, similar to other articles [32,33,34]. The peaks of CIP, both those from the samples with pure CIP (standards) and those corresponding to the composite, were found at the same retention time (2.9 min). The wavelength corresponding to the maximum absorption was also the same for all samples (280 nm), which certifies the purity of the obtained peak [35]. In a previous study, we obtained a lower CIP encapsulation efficiency of 9.3% for PLGA-CIP nanoparticles synthesized using the water-in-oil-in-water (W/O/W) double emulsion method [36]. This may be determined by the limited water solubility of ciprofloxacin. In this study, a high loading efficiency was obtained for both stirring rates selected with the S/O/W, thus rendering this method as a better choice.

### 4.3. Granulometric Distribution

Both PLGA-CIP nanoparticles (500 rpm, 1500 rpm) displayed a negative zeta potential which is essential in bone regeneration and osteointegration [37,38]. Furthermore, Patil et al. showed that the zeta potential plays an important role in nanoparticle diffusion at a cellular level [39]. Botelho et al. confirmed in a study that including silicone in hydroxyapatite lowers the overall charge on the surface of the crystals, which has been found to enhance the biological activity of apatite [40]. Our results are similar to other studies which have shown that a material that has an electronegative surface charge is more accessible for osteoblast attachment and proliferation because negatively charged species (mesenchymal cells and osteoblasts) are attracted to DDSs when put into contact [41].

Particle size plays an important role in their diffusion into cells [42]. The diffusion of nanoparticles into cells was found to increase as particle size decreases. This phenomenon occurs up to a nanoparticle size of 100 nm, since below this size, the cells no longer manage to internalize the particles. Particle size also affects drug release. Smaller particles have a larger specific surface; therefore, almost the entire quantity of the encapsulated drug will be exposed to the surface, leading to a faster release, as opposed to bigger particles where the drug diffuses slowly. Moreover, smaller particles have the disadvantage of aggregating. In a previous experimental design study, we evaluated the relation between several factors such as stirring rate, PLGA concentration, and CIP quantity in order to optimize encapsulation efficiency and nanoparticle size [43]. Furthermore, we investigated the relation between polymer concentration, stirring rate, and drug concentration in several other studies [44,45]. The eleven experiments performed showed that the encapsulation efficiency was affected by the PLGA concentration (PLGA concentration increases; therefore, the loading efficiency increases). The interaction between PLGA concentration and stirring rate also had a positive influence [43,44,45].

The studies showed that the main factors that affected the nanoparticle size of PLGA were PLGA concentration and stirring rate. The nanoparticle size was mainly influenced by the stirring rate in a negative manner (as the stirring rate increases, the nanoparticle size decreases). We concluded that it is recommended to increase the PLGA concentration and lower the CIP quantity at high stirring rates in order to obtain a high encapsulation efficiency and lower-size particles [43,44,45].

### 4.4. Morphological Aspects of PLGA-CIP

The spherical morphology of PLGA-CIP is a direct result of the process of emulsifying the organic phase of the polymer (oil) into an aqueous phase, followed by solvent evaporation [46,47], which leads to the formation of nanoparticles. Key factors such as the choice of solvent, surfactants, polymer concentration, and stirring speed can slightly influence the surface smoothness and size of the nanoparticles but do not alter their overall spherical shape [48].

### 4.5. In Vitro Release Profile

The homogeneity of the structure and the properties of the particles determine the incorporation of CIP on the surface or inside the matrix. Moreover, the structure modulates the release profile from the initial transfer phase (controlled by the surface) [49]. We evaluated the capacity of several formulations to prolong the release of CIP. The control sample containing CIP and hydroxyapatite showed a complete release of the drug in 24 h. The results showed that both PLGA-CIP samples and PLGA-CIP:HA samples exhibited a divided release profile. The first part of the curb, a burst release, corresponds to the first three days. For all samples, at least 60% of CIP incapsulated is released in this stage. The drug release follows a case-I-type behavior.

Burst release was observed to be a stage that characterizes prolonged release systems. This is determined by either the dissolution of CIP adsorbed on the surface of the polymer or the diffusion of the drug situated right below the surface. The drug is then released following a case-0-type behavior that characterizes prolonged release systems [50,51]. The second stage represents the release of the deep encapsulated drug. As the results show, CIP is released at a constant rate which ensures the presence of the drug in the adjacent area of the implant for an extended period of time. In all samples, CIP was released for up to 45 days following a mixed kinetics.

The biomaterial that acts as a carrier for the therapeutic agent must have the ability to incorporate, retain, and progressively deliver the drug to the tissue. We consider our results to show an optimal, local antibiotic delivery system as it ensures a burst release in the first 24 h, followed by a sustained release, in a concentration above the MIC, for several weeks [52,53,54,55]. The results show that the association of hydroxyapatite does not negatively influence the CIP release profile.

In a previous study, we evaluated the release of CIP from scaffolds containing an HA -CIP composite [56]. The CIP release profile is similar to this study, but, in that case, the drug was completely released over a period of 30 days. Furthermore, the present study showed a burst release which rapidly ensures a high local antibiotic concentration. This helps avoid the emergence of drug resistance.

Torshabi et al. showed a CIP release over a period of only 16 days with a burst release on day 8 [57]. Furthermore, according to Thomas et al., CIP release follows a mixed kinetics [58]. Approximately 50% of the encapsulated CIP is released in the first 24 h, which is then followed by a prolonged sustained release.

The results showed a value of n between 0.45 and 0.89, suggesting that both diffusion and erosion guide the release of CIP from the scaffolds. Furthermore, the release profile of CIP from Figure 7 suggests that the release of CIP is not affected by the inclusion of hydroxyapatite into the scaffolds.

### 4.6. Antibacterial Activity

The CIP-PLGA nanoparticle scaffolds exhibited a zone of inhibition with a diameter of 32 mm for Staphylococcus aureus and 24 mm for methicillin-resistant Staphylococcus aureus. This suggests that both germs are highly susceptible to the scaffolds. The encapsulated CIP gradually inhibited the bacteria’s growth. At 24 h when the zones of inhibition were read, only 30% CIP was released from the scaffolds, according to the release profile data. The results confirm that after including CIP into PLGA nanoparticles, drug efficiency against bacteria is maintained.

## 5. Conclusions

Local drug delivery systems continue to represent an option for improving the efficiency and safety of therapeutic agents that, if administered using conventional routes, may exhibit solubility and toxicity problems. The antibacterial activity study showed that all analyzed samples inhibited the growth of the tested bacterial strains, thus confirming the drug release from the biocomposite. Localized ciprofloxacin delivery from PLGA-CIP to a particular site in the biological system (bone in our case) is considered a good method for ciprofloxacin delivery to ensure the desired pharmacological effect. It can release antibiotics directly at the infection site. This implantable system bypasses the limitations of systemic administration, ensuring higher drug concentrations (burst release for the first 24 h followed by prolonged release up to 35 days) at the target site with minimal systemic toxicity. Furthermore, nanoparticle size and zeta potential are optimal for cell diffusion and osteointegration. Further in vivo studies are required to assess the efficiency of these scaffolds.

## Figures and Tables

**Table 1:** MAPLE deposition conditions.

Target	1% Dispersion of PLGA-CIP in 99% (5 mL) n-Hexane, Homogenized Using an Ultrasonic Bath
Laser energy	250 mJ@Hz
Chamber energy	100 mJ @Hz
Laser spot	34 mm^2^
Laser fluence	0.3 J/cm^2^
Target-substrate distance	5 cm
Pressure	1.5 × 10^−2^ mbar
Target rotation speed	50 Hz
Substrate rotation speed	30 Hz
Number of pulses	97890
Substrate type	7 × Ti
Observations	Titanium disks were weighed before and after deposition. After deposition, the discs were stored in a refrigerator at 4 degrees Celsius for further use

**Table 2:** Average values of the inhibition zone diameters obtained by applying the disk diffusion agar method (* resistant, ** intermediate, *** sensitive), DZI—average value of three determinations for diameter of inhibition zone (mm).

	*Sample*	*Staphylococcus aureus*	*Methicillin-Resistant Staphylococcus aureus*
DZI (mm)	Control sample (CIP and HA)	39 ***	27 ***
	PLGA-CIP nanoparticle scaffolds (1500 rpm)	32 ***	24 ***
	Implantable PLGA-CIP LRS	22 ***	13 *
	CIP deposited on Ti disks	27 ***	17 **

## Data Availability

Data is contained within the article and Supplementary Material.

## Supplementary Materials

The following supporting information can be downloaded at: https://www.mdpi.com/article/10.3390/polym16213064/s1, Figure S1. CIP chromatogram obtained for (A) PLGA-CIP (1500 rpm) and (B) PLGA-CIP (500 rpm); Figure S2. Zeta potential of (A) PLGA-CIP (1500 rpm) and (B) PLGA-CIP (500 rpm).
