# Supplementary material for: Development of Hybrid Implantable Local Release Systems Based on PLGA Nanoparticles with Applications in Bone Diseases
Source: Polymers (Basel). 2024 Oct 31;16(21):3064. doi: 10.3390/polym16213064 (PMC11548050; doi:10.3390/polym16213064)
Supplement: Supplementary file 1 [file polymers-16-03064-s001.zip › polymers-3241885-supplementary.pdf]

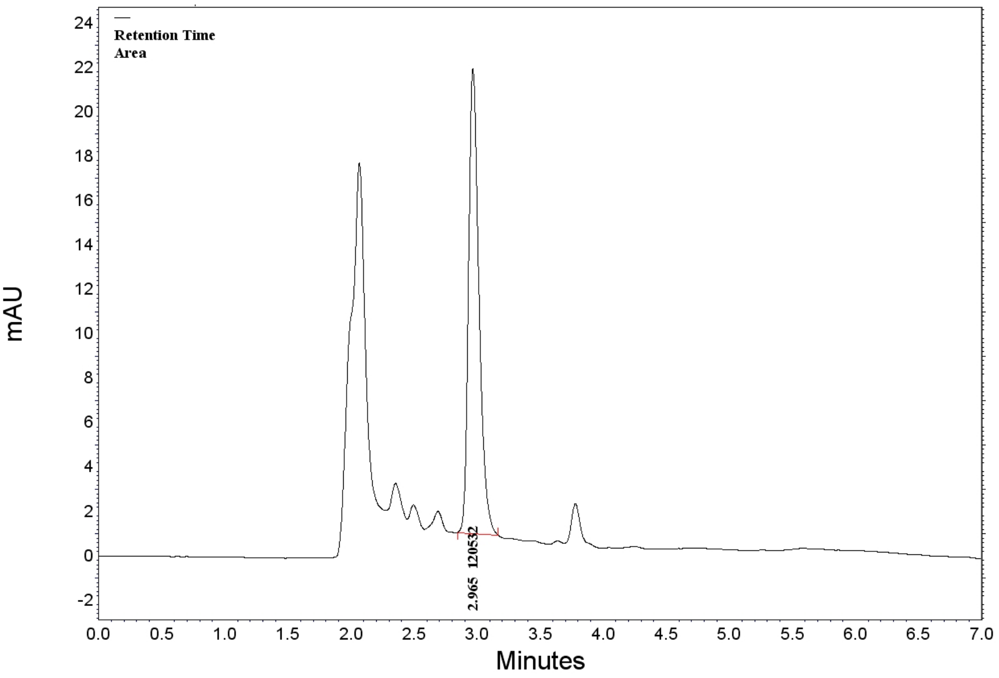

A

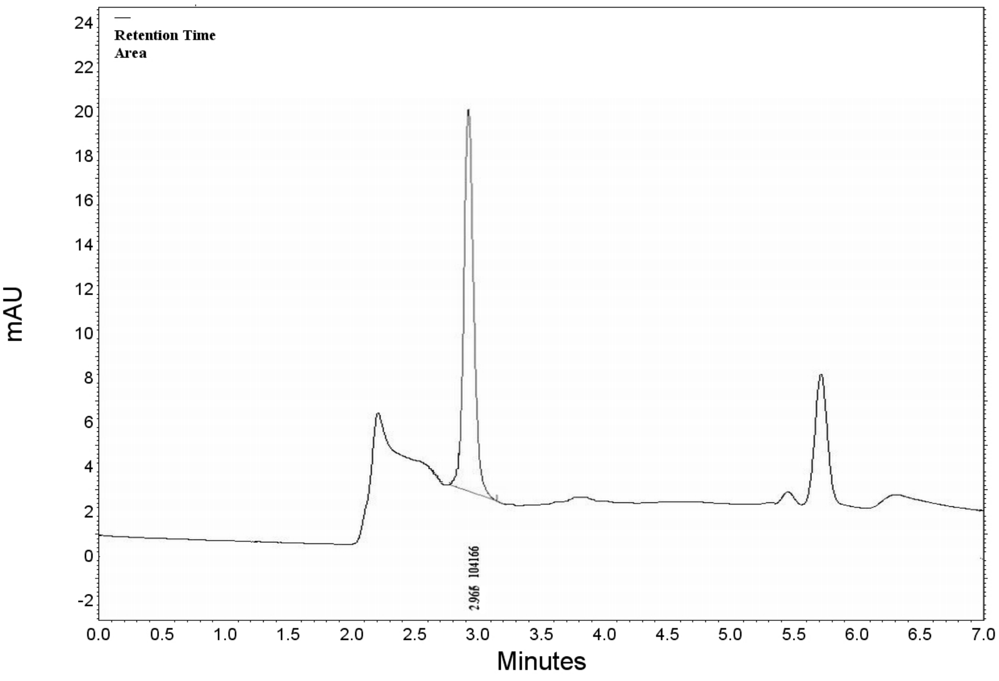

B

Figure S1. CIP chromatogram obtained for A) PLGA-CIP (1500 rpm) and B) PLGA-CIP (500 rpm)

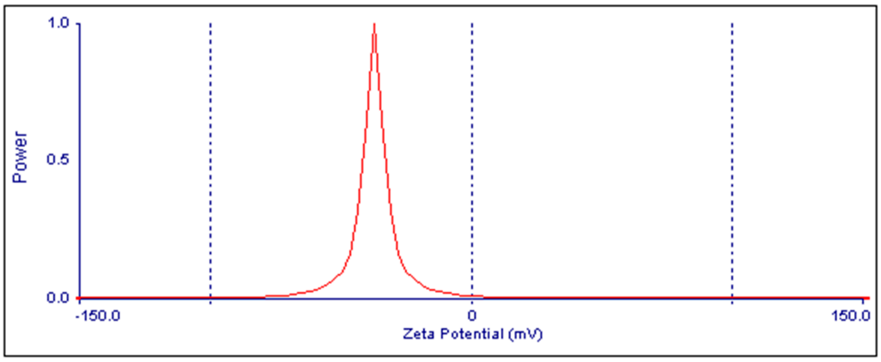

Run	Zeta Potential (mV)	Half Width (mV)
1	-31.03	7.59
2	-38.90	9.14
3	-43.11	9.11
4	-50.19	9.57
5	-37.19	4.15
Mean	-40.08	7.91
Std. Error	3.19	1.00

A

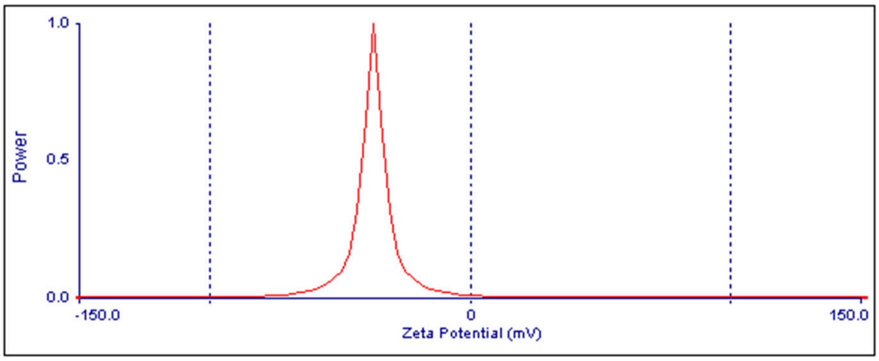

Run	Zeta Potential (mV)	Half Width (mV)
1	-31.03	7.59
2	-38.90	9.14
3	-43.11	9.11
4	-50.19	9.57
5	-37.19	4.15
Mean	-38.04	6.65
Std. Error	0.86	2.50

B

Figure S2. Zeta potential of A) PLGA-CIP (1500 rpm) and B) PLGA-CIP (500 rpm)
